# Monte Carlo simulation of the Varian TrueBeam flattened-filtered beams using a surrogate geometry in PRIMO

**DOI:** 10.1186/s13014-024-02405-w

**Published:** 2024-01-25

**Authors:** Miguel Rodriguez, Josep Sempau, Lorenzo Brualla

**Affiliations:** 1 Hospital Paitilla, Calle 53 y ave Balboa, Panamá, Panama; 2 Instituto de Investigaciones Científicas y Servicios de Alta Tecnología-AIP (INDICASAT-AIP), Ciudad del Saber, Edificio 219, Panamá, Panama; 3 Department of Physics, Universitat Politècnica de Catalunya, Diagonal 647, 08028 Barcelona, Spain; 4 Institut de Tècniques Energètiques, Universitat Politècnica de Catalunya, Diagonal 647, 08028 Barcelona, Spain; 5 Centros de Investigación Biomédica en Red en Bioingeniería, Biomateriales y Nanomedicina (CIBER-BBN), Diagonal 647, 28029 Madrid, Spain; 6 Westdeutsches Protonentherapiezentrum Essen (WPE), Hufelandstraße 55, 45147 Essen, Germany; 7 West German Cancer Center (WTZ), Hufelandstraße 55, 45147 Essen, Germany; 8 Medizinische Fakultät, Universität Duisburg-Essen, Hufelandstraße 55, 45147 Essen, Germany; 9 German Cancer Consortium DKTK, Hufelandstraße 55, 45147 Essen, Germany

**Keywords:** Monte Carlo, Phase space, PRIMO, Linear accelerator

## Abstract

**Background:**

Monte Carlo simulation of radiation transport for medical linear accelerators (linacs) requires accurate knowledge of the geometrical description of the linac head. Since the geometry of Varian TrueBeam machines has not been disclosed, the manufacturer distributes phase-space files of the linac patient-independent part to allow researchers to compute absorbed dose distributions using the Monte Carlo method. This approach limits the possibility of achieving an arbitrarily small statistical uncertainty. This work investigates the use of the geometry of the Varian Clinac 2100, which is included in the Monte Carlo system PRIMO, as a surrogate.

**Methods:**

Energy, radial and angular distributions extracted from the TrueBeam phase space files published by the manufacturer and from phase spaces tallied with PRIMO for the Clinac 2100 were compared for the 6, 8, 10 and 15 MV flattened-filtered beams. Dose distributions in water computed for the two sets of PSFs were compared with the Varian Representative Beam Data (RBD) for square fields with sides ranging from 3 to 30 cm. Output factors were calculated for square fields with sides ranging from 2 to 40 cm.

**Results:**

Excellent agreement with the RBD was obtained for the simulations that employed the phase spaces distributed by Varian as well as for those that used the surrogate geometry, reaching in both cases Gamma ($2\%$, 2 mm) pass rates larger than $99\%$, except for the 15 MV surrogate. This result supports previous investigations that suggest a change in the material composition of the TrueBeam 15 MV flattening filter. In order to get the said agreement, PRIMO simulations were run using enlarged transport parameters to compensate the discrepancies between the actual and surrogate geometries.

**Conclusions:**

This work sustains the claim that the simulation of the 6, 8 and 10 MV flattening-filtered beams of the TrueBeam linac can be performed using the Clinac 2100 model of PRIMO without significant loss of accuracy.

## Background

The Monte Carlo method for radiation transport has been widely used for simulating clinical beams produced by medical linear accelerators (linacs). The model of the beam obtained with this method is usually a collection of particle data, a so-called phase space. A phase space is commonly stored in a file and used for the simulation of further transport in the patient-dependent part of the linac geometry and for the subsequent estimation of the absorbed dose distribution.

Apart from the influence of the approximations adopted in the transport physics, the accuracy of a beam representation obtained by the Monte Carlo method relies on the precise knowledge of the geometrical description and material composition of the linac head, information provided by the machine manufacturer.

The geometrical description of the Varian TrueBeam (TB hereafter) linac has not been disclosed by the manufacturer (Varian, Palo Alto, USA). The options that remain available for obtaining a beam model useful as input to a Monte Carlo dose estimation algorithm are either to create a virtual source model or to use phase-space files (PSF) tallied by the manufacturer. Virtual source models are in general considered a less accurate representation of a beam than a phase space. The main limitation of the PSFs distributed by the manufacturer is that they represent a typical beam which might deviate from the actual beam of a particular linac. In some cases, other limitations could be: (1) a large latent variance [1] that renders the PSF inadequate for obtaining the desired level of statistical uncertainty in a simulation; and (2) the approximations adopted in the Monte Carlo radiation transport code used to produce the phase space. Although the PSFs distributed by Varian for the TB prove to be adequate for most clinical applications, it is also true that dosimetry-relevant problems, such as, the determination of ionization chamber correction factors, demand for highly accurate simulations with subpercentage statistical uncertainties in very small tallying volumes [2]. This type of problems cannot be tackled with the PSFs distributed by the vendor.

Phase spaces distributed by Varian were tallied by performing the radiation transport in a TB geometry generated from the manufacturer blueprints. The simulations were done with geant4 v4.9.2.p01 [3, 4] using the *Standard* electromagnetic model (EM) for the transport physics [5]. The parameters of the initial electron beam were obtained from the simulation of the linac waveguide. These phase spaces are expected to produce dose distributions in water that match the RBD which is a set of commissioning measurements made for a beam considered standard or average. Therefore, they are supposed to closely reproduce the beams of most installed machines. Deviations from dose profiles produced by actual beams are expected to be small but they are unknown *a priori*. For that reason, the manufacturer recommends a customized commissioning of clinical treatment planing systems instead of using the RBD.

In a previous work [6], we proposed a *ad hoc* geometry that proved useful in simulating the flattening-filter-free beams of a TB machine. That geometry was obtained from modifications made to the geometry of the Clinac 2100. The present work is devoted to investigate the feasibility of using the unmodified geometry of a Clinac 2100 (CL21 hereafter) to simulate the flattening-filtered (FF) beams of a TB linac.

## Methods

### Monte Carlo simulations

According to Varian, the geometry of the TB and the CL21 are identical, for Monte Carlo simulation purposes, dowstream from the height of the collimating jaws. Everything located upstream of the jaws in the TB remains undisclosed. The feasibility of simulating the FF beams of a TB with a CL21 geometry was approached in this work: (1) by comparing the energy and angle distributions of the Varian’s published phase spaces for the TB (hereafter simply referred to as TB PSFs) with those simulated in this work in a CL21 geometry (hereafter CL21 PSFs) for the 6, 8, 10 and 15 MV FF photon beams; and (2) by comparing dose distributions from the RBD in water with those obtained using Monte Carlo simulations that started from either the TB PSFs or the CL21 PSFs.

The RBD contains depth dose distributions, as well as in-line and cross-line centered lateral profiles at different relevant depths in water for squared field sizes ranging from $3\times 3$ cm^2^ up to $40\times 40$ cm^2^, for nominal photon energies ranging from 4 MV up to 15 MV.

All the simulations and analyses were done using the PRIMO software version 0.3.1.1772 [7]. PRIMO is a system intended for the simulation of linac beams and the estimation of absorbed dose distributions using the Monte Carlo method for radiation transport. This system allows selecting the Monte Carlo code penelope (version 2011) [8] or a customized version of the fast Monte Carlo code DPM [9, 10] and includes geometrical descriptions for most Varian linacs. It also allows the use of imported phase spaces as particle sources. The software is freely distributed through the web site http://www.primoproject.net.

### Phase spaces

The TB PSFs (version 2) for the 6, 8, 10 and 15 MV FF photon beams, tallied at 26.70 cm from the target, were obtained from the web site http://www.myvarian.com/montecarlo available to Varian’s customers. The CL21 PSFs were created in simulations run with PRIMO using penelope as the Monte Carlo engine and were tallied at a plane located 25.45 cm from the photon target, just upstream the collimation jaws. That is the maximum distance at which the phase-space plane can be located in our geometrical model of the CL21 without colliding with the jaws when they are at maximum aperture. The influence of the additional 1.25-cm-thick air layer on the photon energy and angular distributions obtained from the TB PSFs was neglected.

The parameters of the initial electron beam used in CL21 simulations, namely the energy ($E_0$), the standard deviation of the energy Gaussian function ($\sigma _E$) and the beam divergence ($\epsilon$), were the same as those used for the simulation of the TB PSFs obtained from the header files. The only exception was a slight modification introduced in the standard deviation ($\sigma _R$) of the Gaussian function defining the spatial distribution of the initial electrons. In this work, a circular Gaussian function was used instead of the elliptical one employed for creating the TB PSFs. The value of $\sigma _R$ was the average of the standard deviations on the *x*- and *y*-axis of the elliptical Gaussian function. This approach introduced a maximum deviation in $\sigma _R$ of $40\,\upmu$m with respect to the one used by Varian.

The parameters of the initial beam are detailed in Table 1. C1 and C2 are the electron average angular deflection and the maximum fractional energy loss allowed in one step, respectively, and were chosen according to the beam nominal energy. The rest of penelope transport parameters are as follows: WCC$=200$ keV and WCR$=50$ keV are the energies that separate hard from soft events for inelastic and bremsstrahlung interactions, respectively; EABS($e^{\pm }$)$=$WCC and EABS($\gamma )$ $=$WCR are the cutoff energies at which simulation is terminated and the particle remaining energy is locally absorbed. The reader is referred to the penelope manual [8] for a more detailed description of these parameters. The number of histories simulated was $3\times 10^8$ in all cases. The splitting-roulette variance-reduction technique was applied in the photon target [11].

In addition to the values of C1 and C2 reported in Table 1, a set of simulations for the 6 MV beam was conducted using C1$=$C2$=10^{-3}$ for better accuracy. The rationale for this choice is discussed in Sect. 3.

**Table 1:** Parameters of the initial electron beam used in the simulations of the CL21 with penelope. The values of the penelope  transport parameters C1 and C2 used for the condensed simulation of electrons in the target are also shown

Beam	$\mathbf{E}_{\mathbf{0}}$ (MeV)	${\varvec{\sigma}} _\mathbf{E}$ (MeV)	$\sigma _\mathbf{R}$ (cm)	${\varvec{\epsilon}}$ (mrad)	C1 = C2 (MeV) ($10^{-3}$)
6	6.18	0.053	0.072	1	20
8	8.74	0.074	0.089	1	9
10	10.7	0.091	0.085	1	8
15	13.5	0.115	0.061	1	2

See the text for a description of the parameters

Energy distributions were calculated in seven rings using a radial interval of 1 cm and also in a circular region of 6 cm of radius centered at the central axis (CAX). Angular distributions were calculated in this circular region only. Radial distributions of the planar energy fluence were calculated for all PSFs up to a radius of 5.0 cm. The radial bin size was 0.2 cm.

The percentage of agreement (PA) [12] was used to compare the CL21 and TB energy distributions. For the purposes of this work the PA is formulated as

1 $$\begin{aligned} \texttt{PA}=100 \left[ 1-\frac{\delta }{\texttt{max}(A_\texttt{TB},A_\texttt{CL})}\right] , \end{aligned}$$

where $A_\texttt{TB}$ and $A_\texttt{CL}$ are the area under the TB and CL21 energy distributions, respectively and $\delta$ is the absolute value of the difference of those distributions.

**Fig. 1:**
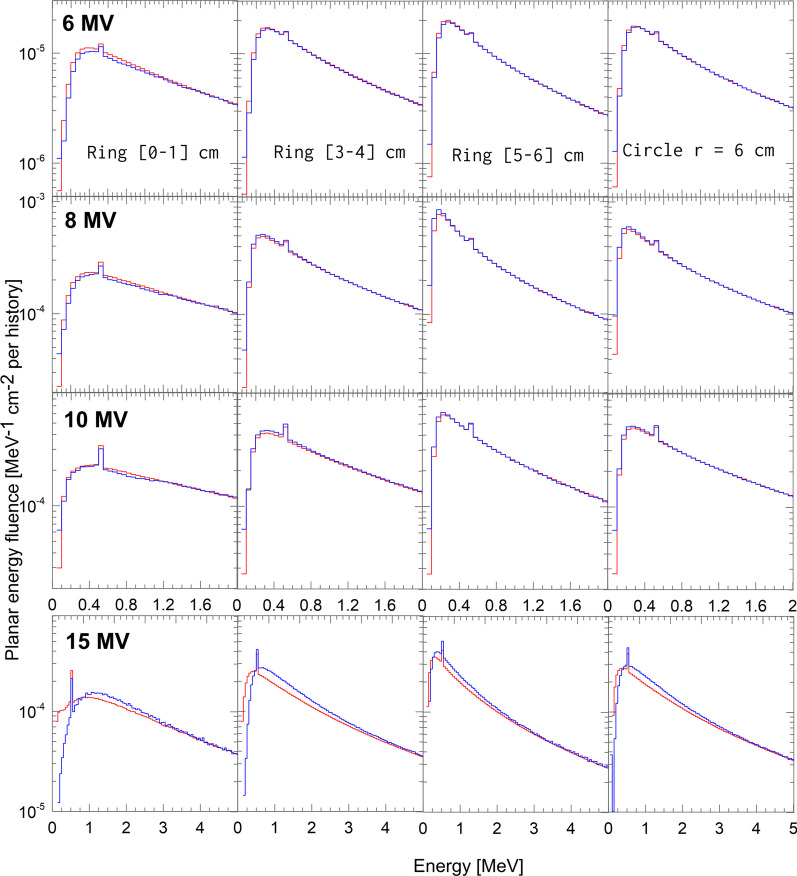
Energy distributions calculated for the TB (red) and CL21 (blue) phase spaces in rings located at three representative distances from the CAX and for a circular region with a radius of 6 cm. Only the energy interval in which the differences between the spectra are more noticeable is represented

### Dose distributions

Dose distributions were estimated in a water phantom for the CL21 and TB by using the corresponding PSFs as source of particles at a source-to-surface distance (SSD) of 100 cm and for square fields 3, 6, 10, 20, and 30 cm of side. The phantom dimensions were set according to the field size and ranged from $10\times 10\times 40$ cm^3^ to $60\times 60\times 40$ cm^3^. The bin size was set to 0.15 cm in the direction of interest (e.g. depth in the case of a depth dose profile) and it was variable in the other directions to a maximum of 0.5 cm. Simple particle splitting [8] with a factor of 300 was applied in the water phantom. DPM was selected as the Monte Carlo engine in all cases. The number of histories simulated was set as to obtain average statistical standard uncertainties of the dose of less than 0.4%. Voxels included in the uncertainty averaging were those with a dose higher than half the maximum dose.

The comparison of the TB and CL21 dose distributions with the RBD was made by gamma analysis [13]. The criteria selected were 2%, 2 mm. Gamma pass rate (GPR_2%,2mm_) was calculated as the percentage of voxels passing the analysis, *i. e., *those with gamma index $\le 1$. The RBD was taken as the reference dose. Depth dose and crossline profiles were extracted from the Monte Carlo 3D dose distributions by trilinear interpolation at the experimental points. Depth dose curves were normalized to the maximum depth. Crossline profiles were normalized to the dose at the CAX. Output factors were also calculated for the two set of dose distributions for square fields of 2, 6, 10, 15, 20, 30 and 40 cm of side at a depth of 10 cm.

## Results

### Phase spaces

Energy distributions calculated in rings with radial intervals [0–1], [3–4] and [5–6] cm and in the circular 6 cm-radius region are shown in Fig. 1. Energy intervals where differences between distributions are negligible were excluded from the graphs for better visualization. Table 2 shows the calculated values of the percentage of agreement for both sets of energy distributions. In general, a good match is observed for all regions for the 6, 8 and 10 MV beams. In contrast, large differences were found for the 15 MV beam in all regions. These are noticeable in the distributions shown in Fig. 1. For the [0–1] cm ring and the energy interval of [0.10–0.75] MeV the energy fluence is on average 40% larger for the TB PSF with respect to the CL21 PSF. This discrepancies for the 15 MV beam are also noticeable in the relatively lower values obtained for the percentage of agreement in table 2, suggesting differences in the photon target and in the design of the flattening filter between both linacs for the 15 MV beam.

**Fig. 2:**
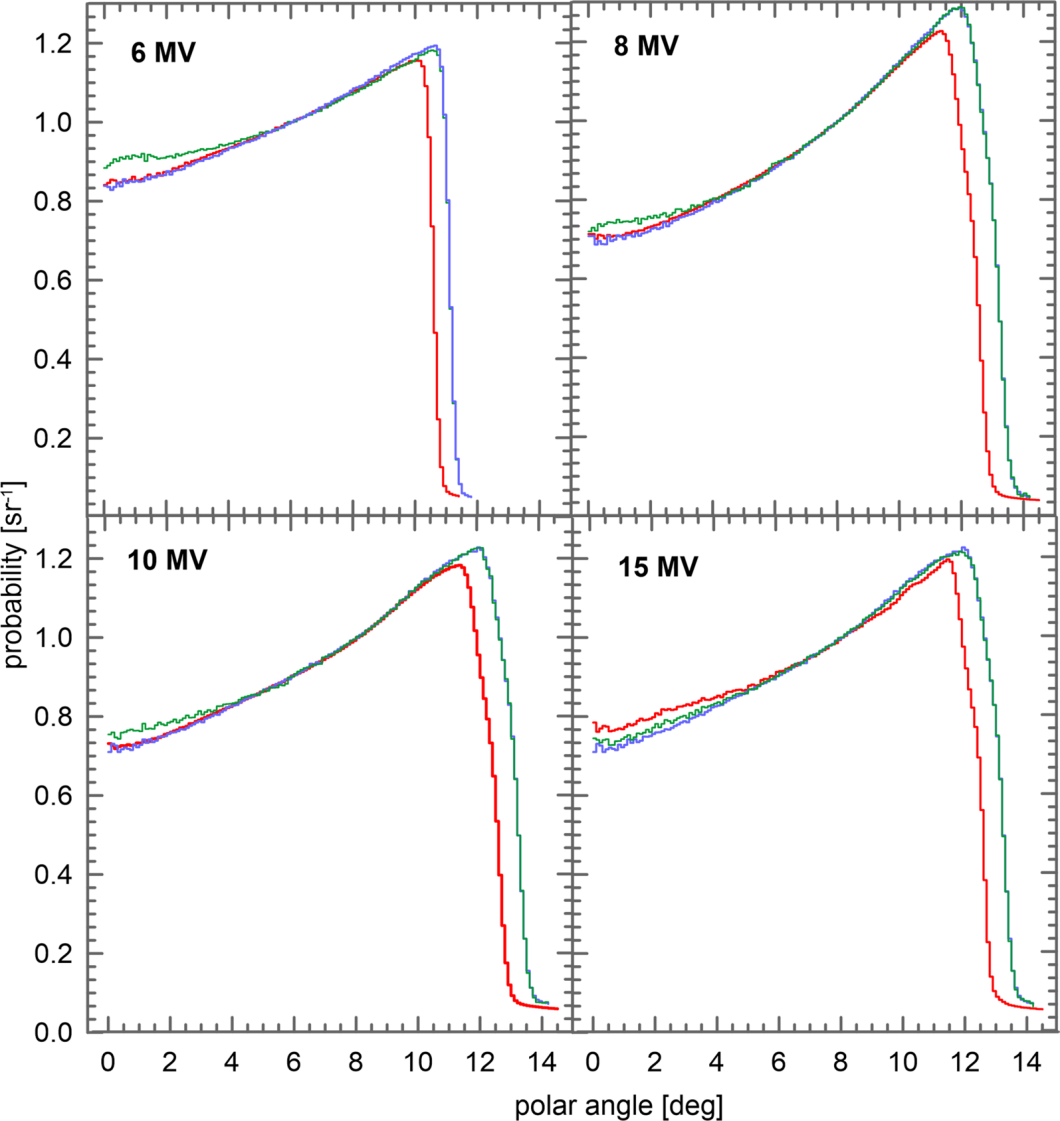
Angular distribution calculated for the TB (red) and CL21 (blue) phase spaces simulated with penelope transport parameters C1 and C2 as specified in table 1. For comparison purposes, the angular distribution obtained for CL21 with transport parameters C1 and C2 equal to $10^{-3}$ is also plotted for each nominal energy (green). Statistical uncertainties are not shown for the sake of better visualization

**Table 2:** Percentage of agreement between the TB and CL21 energy distributions

Beam	Ring interval [cm]						
	[0–1]	[1–2]	[2–3]	[3–4]	[4–5]	[5–6]	[0–6]
6	99.6	99.8	99.8	99.9	99.9	99.8	99.9
8	99.8	99.9	99.9	99.9	99.8	99.6	99.8
10	99.8	99.9	99.9	99.8	99.8	99.6	99.7
15	97.6	97.6	97.5	97.5	97.4	97.5	97.5

Figure 2 shows the angular distribution calculated for both sets of phase spaces. The distributions are expressed per unit solid angle.

Angular distributions discrepancies found for the 15 MV beam are also attributed to differences in the photon target and in the flattening filter. From Fig. 2 it is observed that the CL21 distribution extends to larger angles than the TB distribution for all considered energies. This is due to the design of the secondary shielding block located upstream the jaws which in the geometry of the CL21 was made shorter in the beam direction than specified in the manufacturer’s blueprints to avoid its collision with the upper jaws at large openings. The discrepancies that appear for all nominal energies at large angles, do not have any effect on the absorbed dose distribution on fields smaller than $40\times 40$ cm^2^. Therefore, the only relevant discrepancies appearing in Fig. 2 are those occurring in the 15 MV case.

### Dose distributions

**Fig. 3:**
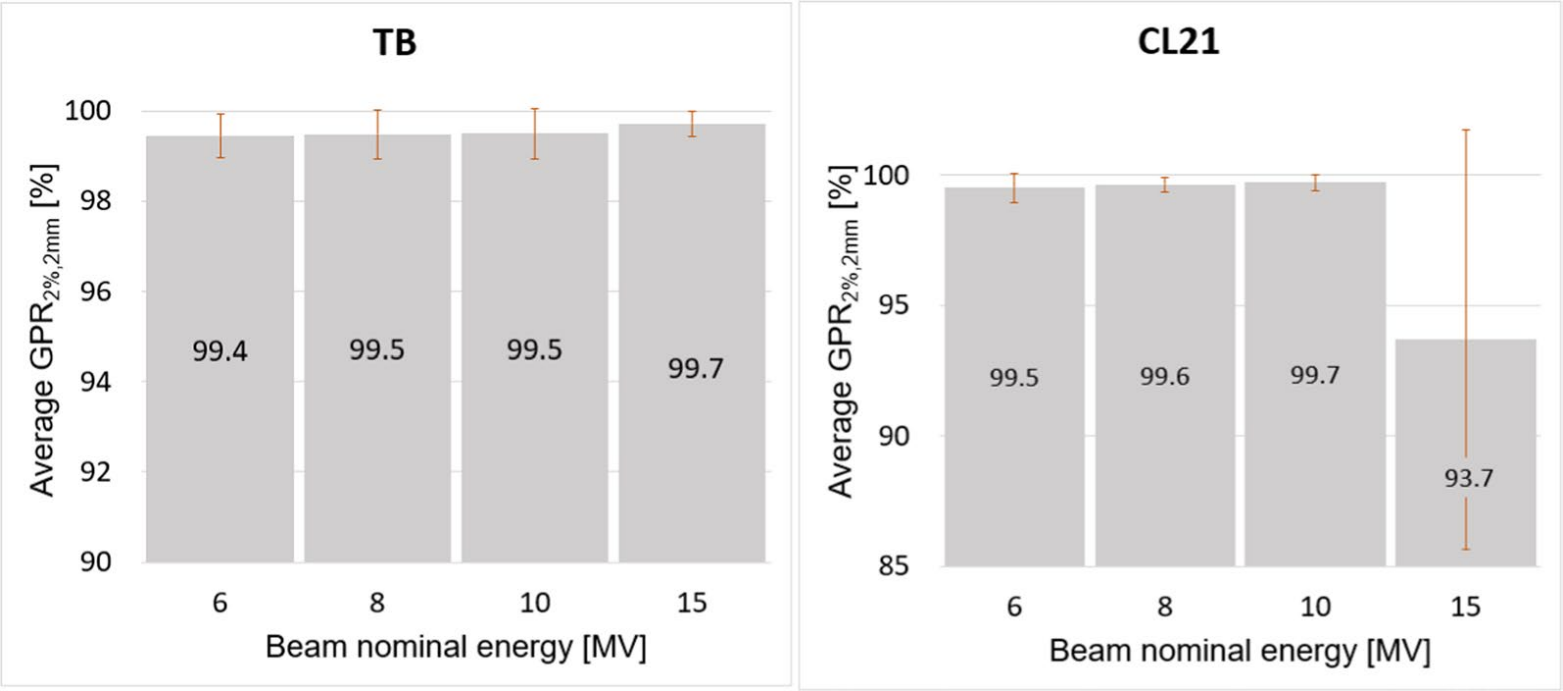
Gamma pass rate for criteria 2%, 2 mm averaged for all fields of a given beam. The standard deviation is also shown

Results of the gamma analysis comparing the TB and CL21 dose distributions with the RBD are shown in Fig. 3. The values of GPR_2%,2mm_ shown in the figure are the average of all fields of a given beam. The standard deviation is also shown in the graphs. A good match is observed for all fields of the TB. For the CL21 good agreement was obtained for the 6, 8 and 10 MV beams with an average GPR_2%,2mm_ larger than 99% and a maximum standard deviation of $0.6\%$. Comparatively, a poorer match is obtained for the 15 MV with an average GPR_2%,2mm_ of only 93.7% and a large standard deviation of 8.0%. The worst GPR_2%,2mm_ ($84.0\%$) for this beam was obtained for the crossline profile of the $30\times 30$ cm^2^ field shown in Fig. 6.

**Fig. 4:**
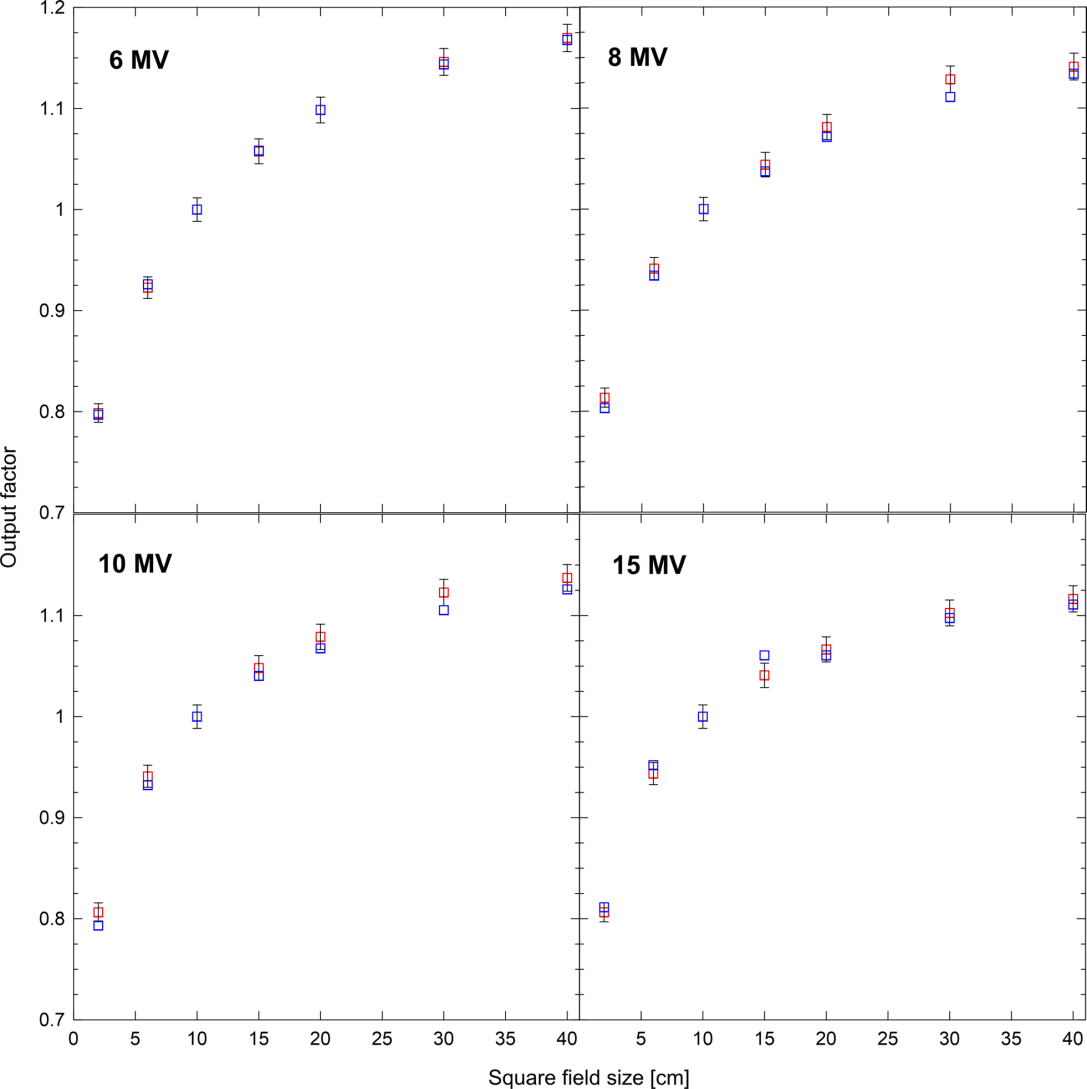
Output factors calculated for the TB (red) and CL21 (blue). For clarity uncertainties are only shown for the TB. For the CL21 uncertainties are similar

**Fig. 5:**
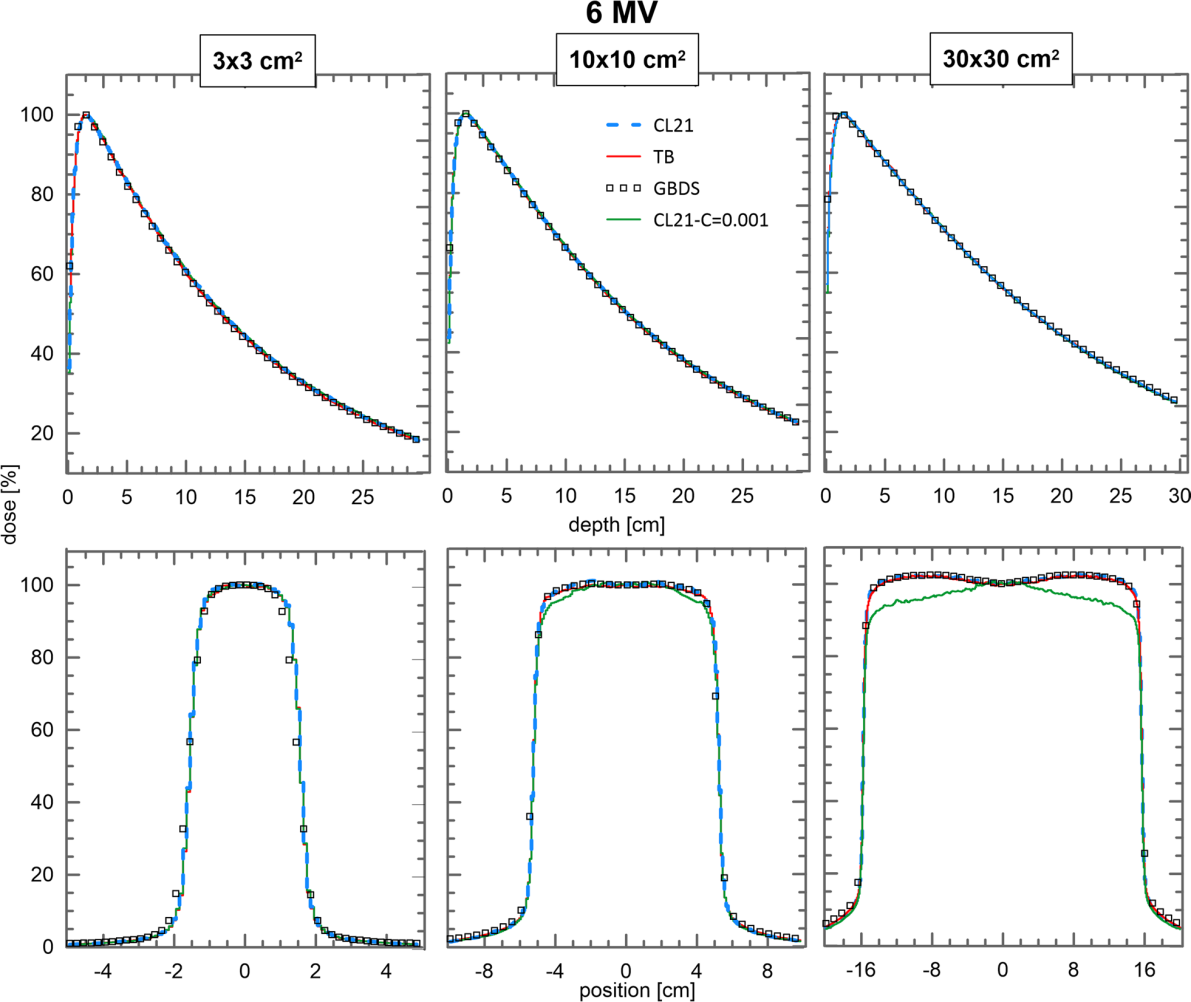
Comparison of depth doses and crossline dose profiles for the $3\times 3$ cm^2^, $10\times 10$ cm^2^ and $30\times 30$ cm^2^ fields for the 6 MV beam of RBD (squares), TB (red), CL21 using the values of C1 and C2 in table 1 (blue) and CL21 using a value of $10^{-3}$ for C1 and C2 (green). Crossline profiles were taken at a depth of 5.0 cm

**Fig. 6:**
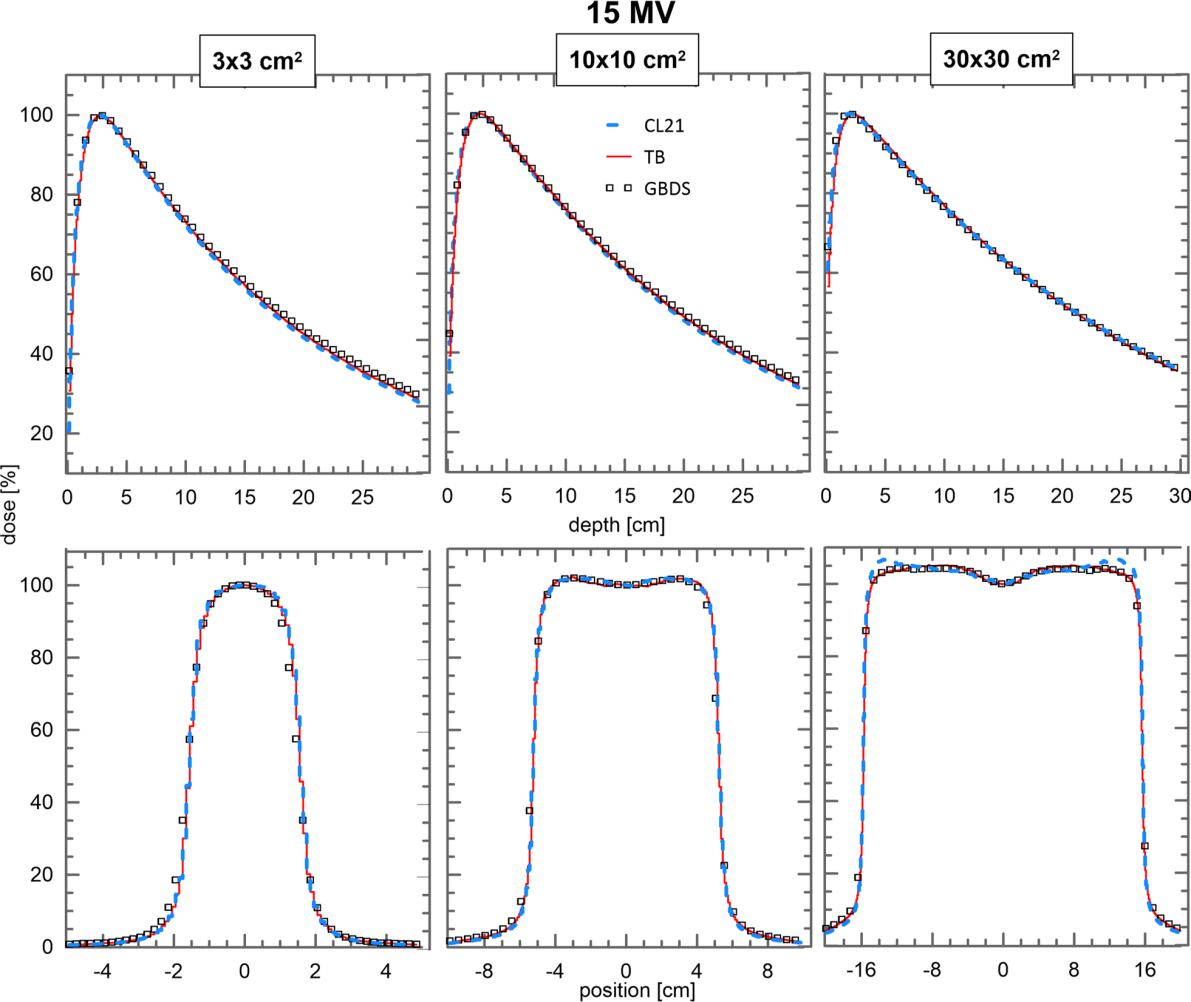
Comparison of depth doses and crossline dose profiles for the $3\times 3$ cm^2^, $10\times 10$ cm^2^ and $30\times 30$ cm^2^ fields for the 15 MV beam of RBD (squares), TB (red) and CL21 (blue). Crossline profiles were taken at a depth of 5.0 cm

Figures 5 and 6 show the comparison of depth dose curves and crossline profiles for the $3\times 3$ cm^2^, $10\times 10$ cm^2^ and $30\times 30$ cm^2^ fields of the 6 and 15 MV beams, respectively. Figure 5 also shows the dose profiles for the CL21 PSFs created by using C1 and C2$=10^{-3}$. An average GPR_2%,2mm_ of $86.5\%$ was obtained in the dose comparison with the RBD for this phase space.

Output factors for the TB and CL21 beams are shown in Fig. 4. Statistical standard uncertainties for both linacs are smaller than $1\%$. A good match is observed for all beams including the 15 MV beam. The relative difference of TB and CL21 output factors is within two standard deviations in all cases.

## Discussion and conclusions

Radiation transport simulation from the TB PSFs downstream to the water phantom with PRIMO yields absorbed dose distributions that are in excellent agreement with the RBD for all considered field sizes. This fact suggests that differences between penelope and geant4 physics models employed for the simulation of the patient-dependent part of the linac and the water phantom are not relevant. Also, possible differences between the geometry of the patient-dependent part coded in PRIMO and the one employed by Varian to validate their PSFs [4], are negligible in terms of the effects produced in the dose distributions.

The simulation of the CL21 linac from the primary electron source downstream to the water phantom, that is, encompassing both the patient-independent and dependent parts of the linac, yields noticeable discrepancies with the RBD. For this simulation, radiation transport parameters in accordance with our previous study on the subject [14] were employed. In particular, C1 and C2 were set to $10^{-3}$, which ensures an accurate angular distribution of bremsstrahlung photons leaving the target. Counterintuitively, to obtain dose distributions that reproduce the RBD for the considered field sizes, it is necessary to employ less stringent values of C1 and C2 (see Table 1), effectively enlarging the electron step length and thus reducing the accuracy of the penelope transport model. This fact suggests that there are relevant differences in the geometry of the patient-independent part of the CL21 and the undisclosed geometry of the TB. The enlarged values of the transport parameters compensate the biased geometry.

The relatively good agreement of energy and angular distributions for the 6, 8 and 10 MV beams is reflected on the dose distributions obtained for those beams when less stringent values of C1 and C2 are employed. Analogously, the larger discrepancies found in the energy and angular distributions for the 15 MV beam, despite the use of less stringent C1 and C2 are also reflected in a poor match of the CL21 dose distributions with the RBD. The cause for these discrepancies could be a change in the material composition of the TB 15 MV flattening filter as it has been suggested previously [15].

Based on our findings, the TB 6, 8 and 10 MV flattening filtered beams can be simulated in the geometry of the CL21 with PRIMO without significant loss of accuracy provided that the transport parameters C1 and C2 are set to the values specified in the present work. This opens the door to dosimetric studies that require a lower statistical uncertainty than that achievable with the PSFs distributed by Varian.

## Data Availability

The data generated by the authors that support the findings of this study are available on request from the corresponding author (LB).

## Abbreviations

CAX: Central axis
CL21: Clinac 2100
DPM: Dose planning method
FF: Flattening filter
GPR: Gamma pass rate
OF: Output factor
PSF: Phase-space file
RBD: Representative beam data
SDD: Source-to-surface-distance
TB: TrueBeam
